# Emerging Molecularly Targeted Therapies in Castration Refractory Prostate Cancer

**DOI:** 10.1155/2013/981684

**Published:** 2013-05-08

**Authors:** Jesal C. Patel, Benjamin L. Maughan, Archana M. Agarwal, Julia A. Batten, Tian Y. Zhang, Neeraj Agarwal

**Affiliations:** ^1^Division of Medical Oncology, University of UT Huntsman Cancer Institute, Salt Lake City, Utah 84112, USA; ^2^Department of Internal Medicine, University of UT, Salt Lake City, Utah 84112, USA; ^3^Department of Pathology and ARUP Laboratories, University of UT, Salt Lake City, Utah 84108, USA

## Abstract

Androgen deprivation therapy (ADT) with medical or surgical castration is the mainstay of therapy in men with metastatic prostate cancer. However, despite initial responses, almost all men eventually develop castration refractory metastatic prostate cancer (CRPC) and die of their disease. Over the last decade, it has been recognized that despite the failure of ADT, most prostate cancers maintain some dependence on androgen and/or androgen receptor (AR) signaling for proliferation. Furthermore, androgen independent molecular pathways have been identified as drivers of continued progression of CRPC. Subsequently, drugs have been developed targeting these pathways, many of which have received regulatory approval. Agents such as abiraterone, enzalutamide, orteronel (TAK-700), and ARN-509 target androgen signaling. Sipuleucel-T, ipilimumab, and tasquinimod augment immune-mediated tumor killing. Agents targeting classic tumorogenesis pathways including vascular endothelial growth factor, hepatocyte growth factor, insulin like growth factor-1, tumor suppressor, and those which regulate apoptosis and cell cycles are currently being developed. This paper aims to focus on emerging molecular pathways underlying progression of CRPC, and the drugs targeting these pathways, which have recently been approved or have reached advanced stages of development in either phase II or phase III clinical trials.

## 1. Introduction

**Figure 1:**
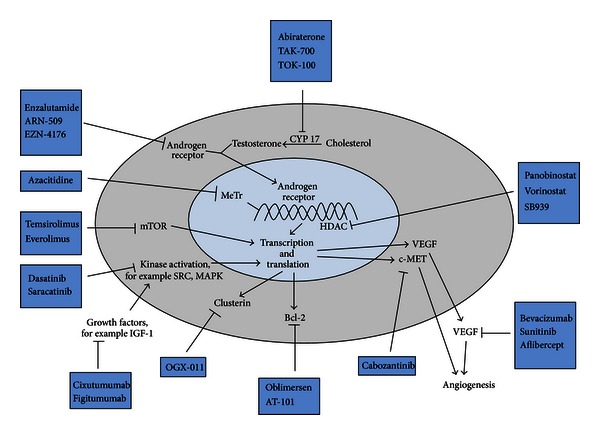
Androgen dependent and independent molecular pathways underlying progression of castration refractory prostate cancer and drugs targeting those pathways. CYP17: Cytochrome P450 17 alpha-hydroxylase and C17,20-lyase; mCRPC: metastatic castration refractory prostate cancer; AR: androgen receptor; OS: overall survival; PFS: progression free survival; c-MET: hepatocyte growth factor receptor; VEGF: vascular endothelial growth factor; MeTr: DNA methyl transferase; HDAC: histone deacetylases; Src: Src kinase; MAPK: MAP kinase; IGF-1: insulin like growth factor-1.

Prostate cancer is the most common noncutaneous malignancy and one of the common causes of cancer related deaths among men in the United States [1]. The majority of men are diagnosed with early stage disease. However, approximately a third will eventually develop metastatic disease. The cornerstone of treatment for advanced disease is medical castration by androgen deprivation therapy with a gonadotropin releasing hormone agonist (GnRH) or less commonly, surgical castration with orchiectomy. Despite initial responses, almost all patients will develop disease progression, a stage known as metastatic castration refractory prostate cancer (mCRPC). One of the reasons why prostate cancer continues to progress is the persistence of androgen receptor signaling, despite castrate level of androgens [2]. Additionally, there are androgen independent pathways responsible for prostate cancer progression. This paper summarizes the recent advancements in the development of therapies that target these molecular pathways in CRPC, with an emphasis on agents that are being evaluated in phase II or III clinical trials and those recently approved for the treatment of CRPC (Figure 1, Tables 1 and 2). 

## 2. Targeting Androgen Signaling Pathway

Several mechanisms have been identified to explain persistent androgen signaling in CRPC [2, 3], including increased AR gene expression, mutation of the AR gene, or upregulation of enzymes involved in androgen synthesis [3–6]. Cytochrome P450 17 alpha-hydroxylase and C17,20-lyase (CYP17) are the critical enzymes for synthesis of androgens in the adrenal glands and prostate tumors [6]. 

Abiraterone acetate, a pregnenolone analog, is an orally administered small molecule that irreversibly inhibits CYP17. Abiraterone was shown to be clinically beneficial and improved overall survival compared to placebo in a landmark phase III trial in patients who had been previously treated with docetaxel (14.8 months versus 10.9 months, *P* < 0.0001) [7]. Abiraterone was well tolerated with mild adverse effects associated with secondary mineralocorticoid excess. In 2011, the FDA approved the use of abiraterone for treatment of CRPC in patients who were previously treated with docetaxel. More recently, another phase III study showed improved radiographic PFS with abiraterone over placebo in men with CRPC who had never received chemotherapy, leading to the regulatory approval of abiraterone in prechemotherapy setting as well [8]. 

Orteronel (TAK-700), like abiraterone, is a novel inhibitor of the CYP17 pathway. However, TAK-700 more specifically inhibits CYP17, 20 lyase versus CYP17 hydroxylase, and does not generally lead to the syndrome of secondary mineralocorticoid excess. The updated data obtained from phase II portion of a phase I/II study of TAK-700 in chemonaive patients with metastatic CRPC were reported in 2012 Genitourinary Cancers Symposium [9]. Ninety-six chemonaive patients with metastatic CRPC were treated in four TAK-700 dose cohorts. The PSA reduction rates were favorable across all cohorts. Of the 51 patients who had RECIST-evaluable disease, 10 had a partial response, 22 had stable disease, and 15 had disease progression. In a phase II study of patients with nonmetastatic CRPC with biochemical recurrence, 38 patients were treated with TAK-700 at a dose of 300 mg twice daily, without prednisone [10]. Treatment at this dose was feasible with manageable toxicities. After three months of treatment, 16% achieved PSA ≤0.2 ng/Ml, 76% achieved ≥50% decrease, and 32% achieved a PSA reduction of ≥90%. Median time to PSA progression was 14.8 months. Currently, there are separate phase 3 trials evaluating TAK-700 in men with progressive CRPC, who are either chemotherapy naïve or posttreatment with docetaxel (Table 1). 

 Enzalutamide (MDV 3100) is a novel AR antagonist that binds to AR with an eight-times higher affinity than bicalutamide and reduces the efficiency of nuclear translocation of the androgen receptor, DNA binding to androgen response elements, and recruitment of coactivators by the androgen receptor [11]. In contrast to bicalutamide, MDV3100 has no known agonist activity when AR is overexpressed. In a phase III trial of men with CRPC with prior docetaxel therapy, enzalutamide when compared to placebo, significantly improved overall survival (18.4 months versus 13.6 months; *P* < 0.001), leading to its regulatory approval in this setting [12]. Another phase III trial of enzalutamide in chemotherapy naïve men with CPRC is currently ongoing (Table 1). 

ARN-509 is a novel small molecule AR antagonist, is structurally and mechanistically similar to enzalutamide, and impairs AR nuclear translocation, as well as AR binding to DNA. In phase I/II study of men with metastatic CRPC, ARN-509 was well tolerated and showed pharmacodynamic evidence of AR antagonism with promising clinical activity. Phase II dose was determined to be 240 mg per day. The most common treatment related grade 1-2 side effects were fatigue, nausea, and pain [13] (Table 2). The phase II portion of the study included three distinct population of men with CRPC: nonmetastatic treatment-naïve CRPC, metastatic CRPC (treatment naïve), and metastatic CRPC (with prior treatment with abiraterone acetate). The primary endpoint was PSA response rate at 12 weeks according to the Prostate Cancer Working Group 2 Criteria. Preliminary results were reported at the 2013 genitourinary cancer symposium. Among 47 men with non-metastatic CRPC, the 12-week PSA response was 91%, and the time to PSA progression had not been reached [14]. Among 46 men with metastatic CRPC, 26 were treatment naïve, and 21 had prior treatment with abiraterone acetate. At 12 weeks, the PSA response was 88% in the treatment naïve cohort. Notably, 29% men with prior treatment with abiraterone acetate had PSA response after 12 weeks, thus indicating activity of ARN-509 in the subset of men with CRPC that developed resistance to abiraterone acetate [15]. 

 Other promising rationally designed drugs inhibiting AR signaling include TOK-001 (galeterone), EZN-4176, and EPI-001. TOK-001, formerly known as VN/124-1, inhibits prostate cancer growth by multiple mechanisms. In addition to inhibiting CYP17, it directly antagonizes the AR receptor and also downregulates AR protein expression [16]. A phase I/II trial of TOK-001 has been initiated in chemonaive patients with mCRPC (Table 2). EZN-4176, an AR mRNA antagonist, is a locked nucleic acid (LNA) oligonucleotide that downregulates AR mRNA and is currently ongoing development in a phase I trial of men with CRPC [17]. EPI-001 inhibits the N terminal domain of the AR, which confers transcriptional activity, and has the capability to overcome castration resistance associated with a gain of function mutations of the ligand binding domain, and expression of constitutionally active splice variants of the AR. EPI-001 is currently awaiting clinical trial development in [18]. 

## 3. Targeting Androgen Independent Molecular Pathways Implicated in Stromal-Epithelial Crosstalk and in Shaping Tumor Microenvironment

### 3.1. Targeting Src Kinase Signaling

The Src family of nonreceptor protein tyrosine kinases (SFKs) is upregulated in various human malignancies, including prostate cancer and their expression directly correlates with disease progression and metastasis [19, 20]. In addition, Src signaling plays an important role in normal bone turnover, and is essential for normal osteoclast functioning, as well as osteoblast proliferation, and has been implicated in the promotion of bone metastasis in prostate cancer [20]. 

Dasatinib (BMS-354825) is a selective small molecule inhibitor of SFKs and other tyrosine kinases, including Bcr-Abl, Kit, and PDGFR*β*. A recent phase II study of chemotherapy naïve patients with CRPC has shown promising results. Forty-three percent of patients at week 12 and 19% patients at week 24 had radiographically stable disease. Additionally, at week 12, 51% of patients achieved ≥40% reduction in urinary N-telopeptide, while 60% had reduction in bone alkaline phosphatase. Treatment with dasatinib was generally well tolerated. Based on the positive results from a separate phase I/II study, which combined dasatinib with docetaxel, a randomized phase III study comparing docetaxel with dasatinib to docetaxel with placebo in castration resistant prostate cancer was conducted. The results, however, failed to show an improvement in overall survival [21]. Additionally, dasatinib is being tested in two randomized phase II trials in combination with abiraterone and cediranib (VEGF TKI), respectively (Table 2). 

Saracatinib (AZD0530) is an oral non-receptor tyrosine kinase inhibitor targeting Src kinases and has been shown to have activity in orthotopic animal models of CRPC [22]. Two phase II studies of saracatinib in CRPC in chemotherapy naive and postdocetaxel settings, respectively, are underway (Table 1). 

### 3.2. Targeting PI3K/Akt/mTOR Pathway

The phosphatidylinositol 3-kinase (PI3 K)/Akt/mammalian target of rapamycin (mTOR) signaling pathway regulates multiple physiological cell processes, which include metabolism, proliferation, differentiation, survival, migration, and angiogenesis. The lipid and protein phosphatase, PTEN (phosphatase and tensin homology protein), is a key negative regulator of Akt activity. Aberrant expression of PI3K and AKT1 genes or loss of PTEN tumor suppression gene leads to downstream upregulation of mTOR and tumorigenesis. Although, everolimus does not have clear significant single agent activity in CRPC, it holds promise in combination therapy with chemotherapeutics given the fact that it restores sensitivity to chemotherapy as do other drugs within the class [23]. Preliminary results of a phase I/II trial combining docetaxel and temsirolimus have showed acceptable safety and antitumor activity and a phase II expansion cohort is underway [24]. Furthermore, multiple phase II studies of mTOR inhibitors as single agent or in combination with chemotherapeutics or biologic agents are ongoing (Table 2). 

### 3.3. Targeting Vascular Endothelial Growth Factor (VEGF) Pathway

 Angiogenesis and neovascularization are necessary for the growth and metastasis of solid tumors. Vascular endothelial growth factor (VEGF) plays a pivotal role in angiogenesis and neovascularization. There is clinical evidence that the simultaneous use of angiogenesis inhibitors and chemotherapeutic drugs may lead to improved outcomes in patients with CRPC [25–27]. Multiple agents have been tested in phase III trials of men with metastatic CRPC. 

Bevacizumab is a humanized monoclonal antibody that inhibits angiogenesis by neutralizing circulating vascular endothelial growth factor (VEGF). Sunitinib is a tyrosine kinase inhibitor (TKI) which similarly targets VEGF receptor. Aflibercept (VEGF Trap) is a recombinant humanized fusion protein consisting of the VEGF extracellular domains and the Fc portion of human immunoglobulin IgG1. However, none of these agents have shown overall survival advantage in phase III trials of men with metastatic CRPC (Table 1). 

### 3.4. Targeting Insulin Like Growth Factor (IGF) Pathway

 The insulin like growth factor (IGF) pathway, which includes IGF receptor-1 (IGF-1R) and its ligands, IGF-I and IGF-II, not only plays a major role in growth, development, and maintenance of homeostasis in normal cells, but also the proliferation of cancers cells, including prostate cancer cells [28]. Higher levels of circulating IGF-I correlates with increased risk of developing prostate cancer, as well as metastatic disease. Blockade of IGF-IR in combination with chemotherapy leads to chemosensitization in androgen-independent prostate cancer cell lines and improved docetaxel antitumor activity in animal models [28]. 

Figitumumab and cixutumumab are fully human monoclonal antibodies targeting IGF-1R. In a phase Ib trial, a combination of figitumumab and docetaxel was well tolerated in patients with a variety of solid tumors, with three patients with CRPC demonstrating an objective response [28]. Encouraged by these results, a phase II study of this combination has been initiated in the setting of CRPC (Table 2). In a phase II study, chemotherapy naïve men with metastatic CRPC were treated with cixutumumab at two different dosing schemes in both cohorts. Approximately 30% of patients had disease stabilization for ≥6 months [29]. The most common drug-related adverse events were fatigue and asymptomatic hyperglycemia. Phase II studies of cixutumumab, either as monotherapy or in combination with temsirolimus, are ongoing in men with chemotherapy naïve metastatic CRPC (Table 2).

### 3.5. Targeting Bcl-2

Overexpression of Bcl-2 is observed in a significant number of men with CRPC. It plays a key role in the onset of castration refractoriness and contributes to the resistance to radiation and docetaxel therapy [30]. Inhibition of Bcl-2 expression leads to increased apoptosis, as well as diminished proliferation and angiogenesis [31]. 

Oblimersen (Genasense, G3139) is an 18-base synthetic oligonucleotide strand that hybridizes with the first six codons of the Bcl-2 RNA transcript, resulting in degradation by endogenous RNase H and the inhibition of Bcl-2 protein expression [31]. In a multicenter, phase II study men with chemotherapy naïve metastatic CRPC with PSA progression, patients were randomized to receive docetaxel with or without oblimersen [30]. Oblimersen was given on days 1–5 to achieve downregulation of Bcl-2 prior to docetaxel, which was given on day 5. Oblimersen was then continued for two more days after docetaxel to allow for adequate coexposure of the two drugs. However, the primary endpoints of the study (confirmed PSA response rate of >30% and a major toxic event rate <45% in the combination arm) were not met [30]. 

AT-101 (R—gossypol acetic acid) is a polyphenolic compound derived from the cottonseed plant [32]. Acting as a BH3 mimetic, AT-101 inhibits the function of Bcl-2 family member proteins by preventing their binding with proapoptotic proteins and upregulates proapoptotic factors. In a phase II study, men with progressive metastatic CRPC received docetaxel and prednisone with or without AT-101 [33]. Although, overall survival, the primary endpoint, was not improved, a potential benefit was observed in a subgroup of high-risk patients. 

These results suggest that apoptotic failure may result from molecular mechanisms other than Bcl-2 over expression. Men with documented underlying Bcl-2 over expression, as a mechanism of chemoresistance, may be more appropriate candidates for combinatorial regimen employing Bcl-2 targeted therapies. 

### 3.6. Targeting Cytoprotective Chaperone Proteins

Clusterin is a cytoprotective chaperone whose transcription is promoted by the androgen receptor and by heat shock factor-1, a key mediator of carcinogenesis [34]. Over expression of clusterin in prostate cancer has been correlated with progression to CRPC [34, 35]. 

Custirsen (OGX-011) is an antisense oligonucleotide to the clusterin mRNA translation initiation site that potently inhibits clusterin expression and enhances the efficacy of anticancer therapies in vitro and in vivo. In a phase II study, men with metastatic CRPC were treated with docetaxel and prednisone with or without OGX-011. Although, there was no improvement in PSA response (the primary endpoint), overall survival (secondary endpoint) was improved in the OGX-011 arm (23.8 months versus 16.9 months) [34]. Currently, three phase III trials combing OGX-011 with chemotherapeutic agents are ongoing in metastatic CRPC (Table 1).

HSP-90 is another multifaceted molecular chaperone implicated in the progression of prostate cancer by the induction of several upstream signaling pathways, which promote aberrant androgen receptor activation and stabilize the androgen receptor protein. Phase II trials are currently ongoing in metastatic CRPC using HSP-90 inhibitors (Table 2) [36]. 

### 3.7. Targeting Hepatocyte Growth Factor/c-Met Signaling Pathway

Hepatocyte growth factor (HGF) is a potent oncogenic protein, which often acts synergistically with VEGF on endothelial cells, as well as several other cellular signaling pathways, including Ras/MEK pathway and PI3K/AKT pathway [37]. HGF acts through its receptor HGFR, also known as c-Met. The HGF/c-Met pathway is overexpressed in prostate cancer and promotes metastasis. Androgen receptor suppresses c-Met transcription, and c-Met expression is up regulated by castration [38]. 

 Cabozantinib (XL-184) is an inhibitor of c-MET and VEGF receptor tyrosine kinases. In a phase II study of men with metastatic CRPC, treatment with cabozantinib, when compared to placebo, resulted in improved PFS, reduction of soft tissue lesions, resolution of bone scans, and decreased pain [39]. Currently, two phase III trials of cabozantinib are ongoing in men with metastatic CRPC, with prior docetaxel and with prior abiraterone or MDV3100 (Table 1). 

### 3.8. Targeting Epigenetic Pathways

AR, a transcription factor, binds with the androgen response elements (AREs) located throughout the genome and recruits various cofactors, such as histone acetyltransferases (HATs) and histone deacetylases (HDACs). This leads to the formation of multiprotein complexes involved in the AR mediated transcriptional regulation of various genes implicated in prostate cancer growth and proliferation [40]. In prostate cancer, HDAC inhibition leads to decreased proliferation of cell lines and decreased tumor growth in preclinical models [41]. 

Vorinostat is a small molecule inhibitor of class I and II HDACs and has shown promising antitumor activity in prostate cancer cell lines and animal models of prostate cancer. Unfortunately, a phase II trial in patients with mCRPC showed no PSA or objective response and an unacceptable toxicity profile. Panobinostat, a pan-deacetylase inhibitor, has undergone testing in a phase I trial in combination with docetaxel in CRPC [42]. Furthermore, panobinostat is being tested in a phase I/II study in combination with bicalutamide and in a phase II study, as monotherapy for patients with CRPC (Table 2). 

Another epigenetic pathway of interest is DNA methylation. An open chromatin structure induced by hypomethylation facilitates gene transcription, whereas a closed structure inhibits transcription. Azacitidine, an inhibitor of DNA methyltransferase, induces hypomethylation and reverses the silencing of tumor suppressor genes. It has been shown to restore sensitivity to androgen deprivation therapy, as well as chemotherapy in preclinical models of prostate cancer [23]. In a phase II trial of men with chemonaive CRPC progressing on combined androgen blockade, addition of azacitidine significantly prolonged PSA doubling time [43]. Currently, azacitidine is being tested in a phase II trial in combination with docetaxel in CRPC with prior docetaxel therapy (Table 2). 

### 3.9. Targeting Bone Metastasis

 The majority of patients with mCRPC develop osteoblastic bone metastasis accompanied by simultaneous bone destruction, due to increased osteoclastic activity. The receptor activator of nuclear factor-kB ligand (RANKL) is critical for the formation, function, and survival of osteoclasts. In preclinical models of prostate cancer, inhibition of osteoclasts leads to improvement of sclerotic changes in the bones [44]. In a phase III trial, zoledronic acid, an inhibitor of osteoclasts, significantly decreased the incidence of skeletal related events (SRE) and increased the median time to the first SRE, over placebo [45]. 

Denosumab is a monoclonal antibody against RANKL. Compared to zoledronic acid, denosumab improved median time to first SRE in a phase III trial of men with CRPC with bone metastasis (20.7 versus 17.1 months; HR: 0.82; 95% confidence interval, 0.71–0.95; *P* = 0.0002 for noninferiority; *P* = 0.008 for superiority). 

Radium-223 chloride (Ra-223), a radiopharmaceutical agent, targets bone metastasis by emitting high energy alpha-particles of extremely short range (<100 *μ*m). In a phase III, placebo controlled trial of men with symptomatic CRPC with bone metastasis, Ra-223 significantly improved overall survival (14.0 versus 11.2 months, two-sided *P* = 0.00185; HR = 0.695; 95% CI, 0.552–0.875) and delayed time to first SRE (13.6 versus 8.4 months, *P* = 0.00046; HR = 0.610; 95% CI, 0.461–0.807) [46].

### 3.10. Modulating the Immune System

Regulatory approval of sipuleucel-T for mCRPC has validated modulation of the immune system as an effective strategy in prostate cancer. Sipuleucel-T consists of autologous antigen presenting cells enriched for a CD54+ DC fraction that are harvested by leukopheresis and cultured with a fusion protein (PA2024), comprising of prostate acid phosphatase (PAP) and granulocyte-monocyte colony stimulating factor (GM-CSF) [47, 48]. In a phase III trial of men with asymptomatic chemonaïve metastatic CRPC, the median overall survival was significantly improved with sipuleucel-T over placebo (25.8 versus 21.7 months; HR = 0.77; *P* = 0.02), with a relative reduction of 22% in the risk of death in the sipuleucel-T group (HR = 0.78; *P* = 0.03) [48]. Upon disease progression, 64% men in the control arm crossed over to a nonrandomized open-label protocol to receive an investigational autologous immunotherapy made from cryopreserved cells (APC8015F). In an exploratory analysis, after adjusting for the effect of APC8015F and assuming that APC8015F was as effective as sipuleucel-T, the estimated overall benefit with sipuleucel-T was 7.8 months [49]. 

### 3.11. Virus-Based Vaccines

The inherent immunogenicity of viruses and the high level of gene expression seen with viral vectors lead to a strong inflammatory response and may lead to an improved immune response against tumor antigens expressed by viruses [50]. Prostvac-VF utilizes a heterologous prime/boost vaccination strategy. It consists of two recombinant viral vectors (vaccinia vector and fowlpox vector), each encoding transgenes for PSA and TRICOM. TRICOM consists of costimulatory molecules, including ICAM (intercellular addition molecules)-1 (CD54), B7.1 (CD80), and leukocyte function-associated antigen-3 (LFA-3)(CD58) [51]. In a randomized phase II trial of men with chemo-naïve, minimally symptomatic metastatic CRPC, Prostvac-VF extended overall survival by 8.5 months over placebo (25.1 versus 16.6 months, *P* = 0.0061; HR = 0.56; 95% CI, 0.37–0.85) [52]. Ongoing clinical trials of Prostvac-VF in advanced phases include those in the setting of non-mCRPC, in asymptomatic/minimally symptomatic metastatic CRPC and in symptomatic metastatic CRPC (in combination with docetaxel) (Tables 1 and 2). 

### 3.12. Cytotoxic T Lymphocyte Antigen 4 (CTLA-4) Antibody

Cytotoxic T lymphocyte antigen 4 (CTLA-4) is a key negative regulator of T-cell responses, inhibits recognition of self-antigens by T cells, and can downregulate the antitumor immune response. Ipilimumab and tremelimumab are fully human, monoclonal antibodies against CTLA-4. Objective and PSA responses have been described in phase II studies of ipilimumab in prostate cancer [53, 54]. Recently, two phase III trials of ipilimumab in chemonaive- and postdocetaxel metastatic CRPC settings have completed accrual with results expected in the near future (Table 1). 

### 3.13. Radiolabeled Monoclonal Antibody

Monoclonal antibodies (MoAbs) targeting prostate surface membrane antigen (PSMA) are in advanced phases of development. PSMA is a type II membrane glycoprotein, which is markedly upregulated in prostate cancer [55]. Deimmunized murine MoAb J591 (muJ591) has been chosen as a vehicle to deliver radioisotopes, because of its high affinity for PSMA [56]. Among various radioisotopes used with mu J591, 177 lutetium can be administered in higher doses, with comparatively less radiation to the marrow and because of its gamma emission, it enables imaging to be performed using the treatment doses [55]. A randomized, phase II study is currently evaluating efficacy of 177Lu-J591 (versus placebo, i.e., 111 In-J591) in combination with ketoconazole and hydrocortisone in nonmetastatic CRPC (Table 2).

### 3.14. Tasquinimod

Tasquinimod is a quinoline-3-carboxamide analog that possesses antiangiogenic and immunomodulatory properties [57]. It significantly delayed disease progression, compared to placebo, in a randomized phase II trial of minimally symptomatic men with metastatic CRPC. A phase III, placebo controlled, randomized trial of tasquinimod has recently completed accrual with the primary endpoint of progression free survival (Table 1).

## 4. Conclusions

Herein, we have provided a thorough, but selective list of androgen dependent and independent molecular pathways recognized to drive the progression of CRPC. Additionally, we summarized the novel agents targeting these pathways, which have been recently approved or have reached advanced stages of clinical development. With the discovery of increased intratumoral expression of key enzymes in the steroid synthesis pathway, agents such as abiraterone acetate, orteronel, and TOK-001 were developed to further minimize androgen-driven tumor progression. When AR gene amplification and mutations were shown by in situ studies, enzalutamide and ARN-509 were developed to abrogate the actions of AR in facilitating tumor progression. Regulatory approval of sipuleucel-T for minimally symptomatic mCRPC has revolutionized the way immune therapy is used in management of malignancy in general. Development of other promising immune-modulatory agents such as Prostvac-VF, ipilimumab, and tasquinimod have reached phase III trials. Classic pathways common to many malignancies, such as apoptotic pathways (bcl2), src-kinase, p53, and PTEN, are also dysregulated by mutations, gene duplications/deletions, and chromosomal changes. These discoveries have provided the rationale for testing agents targeting these pathways for treatment of CRPC. 

The recent proliferation of therapeutic agents for treatment of metastatic CRPC is indicative of the dramatic changes occurring in the field. Amidst this changing landscape of treatment options for metastatic CRPC, many ongoing trials are likely to result in regulatory approval of newer agents. Advances in evidenced based oncology have led to cost-efficient deep sequencing and individual genome sequencing capability. These and similar tools will give physicians insight into the pathophysiology of the tumors of each individual and help personalize treatment based on the underlying molecular pathway or pathways, driving tumor progression. More rationally designed studies based on predictive biomarkers are needed to guide our way through the changing landscape of treatment and the ever increasing treatment options.

## Figures and Tables

**Table 1:** Selected ongoing and recently concluded phase III trials in castration refractory prostate cancer.

Target	Drug	Trial	Phase	Primary endpoint	Clinical trial identifier number (accessed on January 22, 2013)	Outcome
Androgen dependent pathways						

CYP17	Orteronel (TAK-700)	Randomized, double-blind, orteronel plus prednisone versus placebo plus prednisone in mCRPC with prior docetaxel therapy	III	OS	NCT01193257	Ongoing
	Orteronel (TAK-700)	Randomized, double-blind, orteronel plus prednisone versus placebo plus prednisone in progressive, chemotherapy naive mCRPC	III	OS	NCT01193244	Ongoing
	Abiraterone	Randomized, double-blind, prednisone and abiraterone or placebo and prednisone in mCRPC after failed docetaxel	III	OS	NCT00638690	Improved OS with abiraterone
	Abiraterone	Randomized, double-blind, prednisone and abiraterone or placebo and prednisone in chemotherapy naive mCRPC	III	PFS	NCT00887198	Improved radiographic PFS with abiraterone

AR	Enzalutamide (MDV 3100)	Randomized, double-blind, MDV3100 versus placebo in chemotherapy naive progressive mCRPC	III	OS, PFS	NCT01212991	Ongoing
	Enzalutamide (MDV 3100)	Randomized, double-blind, MDV3100 versus placebo in mCRPC with prior docetaxel	III	OS	NCT00974311	Improved OS with enzalutamide

Androgen independent pathways						

Src kinase	Dasatinib	Randomized, double-blinded, docetaxel, prednisone, and dasatinib versus docetaxel, prednisone, and placebo in mCRPC	III	OS	NCT00744497	No difference in OS

c-MET	Cabozantinib	Randomized, placebo controlled of mitoxantrone and prednisone versus cabozantinib in mCRPC	III	Pain response	NCT01522443	Ongoing
	Cabozantinib	Randomized, placebo controlled prednisone versus cabozantinib in mCRPC with prior docetaxel and abiraterone or MDV3100	III	OS	NCT01605227	Ongoing

Clusterin	Custirsen (OGX-011)	Randomized, open label with cabazitaxel and prednisone with or without custirsen in second line chemotherapy in mCRPC	III	OS	NCT01578655	Ongoing
	Custirsen (OGX-011)	Randomized, docetaxel and prednisone with and without custirsen in chemotherapy naïve mCRPC	III	OS	NCT01188187	Ongoing
	Custirsen (OGX-011)	Randomized, docetaxel retreatment or cabazitaxel and prednisone with and without custirsen in mCRPC	III	Pain response	NCT01083615	Ongoing

VEGF	Bevacizumab (VEGF antibody)	Randomized, double-blinded, docetaxel and prednisone with and without bevacizumab in mCRPC	III	OS	NCT00110214	No difference in OS
	Aflibercept (VEGF trap)	Randomized, double-blind, aflibercept versus placebo in mCRPC with ongoing docetaxel and prednisone	III	OS	NCT00519285	No difference in OS
	Sunitinib (VEGF tyrosine kinase inhibitor)	Randomized, double-blind, sunitinib and prednisone versus prednisone in mCRPC with prior docetaxel	III	OS	NCT00676650	No difference in OS

Immunotherapy	Prostvac-VF	Randomized Prostvac-VF with and without GM-CSF in chemotherapy naive mCRPC	III	OS	NCT01322490	Ongoing
	Ipilimumab	Randomized ipilimumab versus placebo in chemotherapy naive mCRPC	III	OS	NCT01057810	Ongoing
	Ipilimumab	Randomized ipilimumab versus placebo in mCRPC following radiotherapy with prior docetaxel	III	OS	NCT00861614	Ongoing
	Tasquinimod	Randomized tasquinimod versus placebo in chemotherapy naive mCRPC	III	PFS	NCT01234311	Ongoing

CYP17: Cytochrome P450 17 alpha-hydroxylase and C17,20-lyase; mCRPC: metastatic castration refractory prostate cancer; AR: androgen receptor; OS: overall survival; PFS: progression free survival; c-MET: hepatocyte growth factor receptor; VEGF: vascular endothelial growth factor.

**Table 2:** Selected ongoing and recently concluded phase II trials in castration refractory prostate cancer.

Target	Drug	Trial	Phase	Primary endpoint	Clinical trial identifier number
Androgen dependent pathways					

CYP17	TOK-001 (galeterone)	Single arm, open label, dose escalation, study of TOK-001 in chemotherapy naive mCRPC	I/II	Safety, PSA response (≥50%)	NCT00959959

AR	Enzalutamide (MDV 3100)	Randomized, double-blind, MDV3100 versus bicalutamide in CRPC	II	PFS at 24 months	NCT01664923
	Enzalutamide (MDV 3100)	Randomized, double-blind MDV3100 versus bicalutamide in CRPC	II	PFS at 24 months	NCT01288911
	ARN-509	Open label, single arm, ARN-509 in CRPC	I/II	PSA response	NCT01171898

AR mRNA antagonist	EZN-4176	Open label, single arm study of EZN-4176 in CRPC	I	Maximum tolerated dose, safety	NCT01337518

Androgen independent pathways					

Src kinase	Dasatinib	Randomized, open label of cediranib and dasatinib versus cediranib and placebo in CRPC resistant to docetaxel	II	PFS	NCT01260688
	Dasatinib	Randomized, open label of abiraterone and prednisone with or without dasatinib in mCRPC	II	PFS	NCT01685125
	Saracatinib	Randomized, double-blind, saracatinib versus placebo in mCRPC with prior docetaxel	II	Time to disease progression, PFS	NCT01267266
	Saracatinib	Single arm, saracatinib in mCRPC	II	PSA response	NCT00513071

mTOR	Everolimus (RAD001)	Single arm, RAD001 in mCRPC	II	Biochemical response rate	NCT00629525
	Everolimus (RAD001)	Single arm, RAD001 with docetaxel and bevacizumab in mCRPC	I/II	Safety	NCT00574769
	Everolimus (RAD001)	Single arm, RAD001 with docetaxel in mCRPC	I/II	Safety, objective response	NCT00459186
	Everolimus (RAD001)	Single arm, carboplatin, everolimus, and prednisone in mCRPC with prior docetaxel	II	Time to progression	NCT01051570
	Temsirolimus	Single arm, cixutumumab with temsirolimus in chemotherapy naive CRPC	I/II	Safety, objective response/PSA response	NCT01026623
	Ridaforolimus	Randomized, placebo controlled, bicalutamide with or without ridaforolimus in mCRPC	II	PSA response, dose limiting toxicities	NCT00777959

IGF-1R	Figitumumab (CP-751871)	Randomized, noncomparative, two arm, open label, docetaxel and prednisone with and without figitumumab in mCRPC	II	PSA response, objective response	NCT00313781
	Cixutumumab (IMC-A12)	Single arm, open label, cixutumumab in chemotherapy naive mCRPC	II	Time to progression, pharmacokinetics	NCT00520481
	Cixutumumab (IMC-A12)	Single arm, cixutumumab with temsirolimus in chemotherapy naive CRPC	I/II	Safety, objective response/PSA response, and time to progression	NCT01026623

Bcl-2	Oblimersen	Randomized docetaxel with or without oblimersen in mCRPC	II	PSA response >30%, major toxic event rate <45%^‡^	NCT00085228
	R-(-)-gossypol acetic acid (AT-101)	Randomized, docetaxel and prednisone with or without AT-101 in chemotherapy naive mCRPC	II	OS*	NCT00571675

Hsp90	STA-9090	Single arm, open label, STA-9090 in mCRPC with prior docetaxel	II	PFS at 6 months	NCT01270880

Hsp90	AT13387	Two arm, open-label, parallel group, randomized, AT13387 with or without abiraterone in mCRPC progressing on abiraterone	I/II	Safety and tolerability response per PCWG2	NCT01685268

HDAC	Panobinostat (LBH589)	Single arm, panobinostat in mCRPC with prior docetaxel	II	PFS at 24 weeks	NCT00667862
	Panobinostat (LBH589)	Randomized panobinostat at two dose levels combined with bicalutamide for CRPC	I/II	Safety, dosing schedule, proportion free of progression and without symptomatic deterioration at 9 months	NCT00878436
	Vorinostat	Single arm, vorinostat for mCPRC with prior chemotherapy	II	PFS at 6 months**	NCT00330161
	SB939	Open label, single arm, SB939 for recurrent or mCRPC	II	PFS PSA response	NCT01075308

DNA methyltransferase	Azacitidine	Single arm, docetaxel retreatment, and prednisone with azacitidine in mCRPC with prior docetaxel	I/II	Safety PSA and objective response	NCT00503984

Immunotherapy	Prostvac-VF	Randomized, open label, flutamide with and without Prostvac-VF in non-mCRPC	II	Time to treatment failure	NCT00450463
	Prostvac-VF	Randomized, open label, docetaxel and prednisone with or without Prostvac-VF in mCRPC	II	OS	NCT01145508
	177Lu radiolabeled monoclonal antibody (Ab) HuJ591 (177Lu-J591)	Randomized ketoconazole and hydrocortisone plus 177Lu-J591 or placebo (111In-J591 ) in non-mCRPC	II	Proportion free of radiographically evident metastases at 18 months	NCT00859781

CYP17: Cytochrome P450 17 alpha-hydroxylase and C17,20-lyase; mCRPC: metastatic castration refractory prostate cancer; PSA: prostate-specific antigen; AR: androgen receptor; PFS: progression free survival; OS: overall survival; mTOR: mammalian target of rapamycin; IGF-1: insulin like growth factor-1; Hsp: heat shock protein; HDAC: histone deacetylases.

^‡^Primary endpoints not met.

*Primary endpoint not met.

**Significant toxicities, no one met the primary endpoint.
